# Chemical Profile and Aroma Effects of Major Volatile Compounds in New Mulberry Leaf Fu Brick Tea and Traditional Fu Brick Tea

**DOI:** 10.3390/foods13121808

**Published:** 2024-06-08

**Authors:** Yuezhao Deng, Cheng Li, Yineng Chen, Zhuoyang Zou, Junyao Gong, Chengwen Shen, Kui Fang

**Affiliations:** 1 College of Information and Intelligent Science and Technology, Hunan Agricultural University, Changsha 410128, China; dengyuechao@hnnydx.wecom.work (Y.D.); hnalc@stu.hunau.edu.cn (C.L.); zzy@stu.hunau.edu.cn (Z.Z.); q1@stu.hunau.edu.cn (J.G.); 2 College of Horticulture, Hunan Agricultural University, Changsha 410128, China; scw69@163.com; 3 School of Information Science and Engineering, Hunan Women’s College, Changsha 410000, China; yinengchen@hunau.edu.cn

**Keywords:** mulberry leaf Fu brick tea, volatile compounds, aroma attributes, sensory evaluation, OPLS-DA

## Abstract

This study aimed to investigate the aroma effects of key volatile compounds in a new type of mulberry leaf Fu brick teas (MTs) and traditional Fu brick teas (FTs). Headspace solid–phase microextraction (HS-SPME), gas chromatography–mass spectrometry (GC-MS), sensory evaluation, and chemometrics were used to determine the differences in key flavour qualities between the two. The results showed that a total of 139 volatile components were identified, with aldehydes, ketones, and alcohols dominating. Orthogonal Partial Least Squares Discriminant Analysis (OPLS-DA) combined with the odour activity value (OAV) showed that seven aroma compounds had an OAV > 10, including 2-(4-methylcyclohex-3-en-1-yl) propan-2-ol with floral and fruity aroma and green attributes, 6-methylhept-5-en-2-one, (E)-6,10-dimethyl-5,9-Undecadien-2-one, (3E,5E)-octa-3,5-dien-2-one, Benzaldehyde, and (E)-3,7,11,15-tetramethylhexadec-2-en-1-ol, which were more abundant in MTs than FTs; Cedrol with sweet aroma attributes was more consistent in MTs than FTs, and we suggest that these odour compounds are important aroma contributors to MTs. Taken together, these findings will provide new insights into the mechanism of formation of the characteristic attributes of aroma in MTs.

## 1. Introduction

Fu brick tea is a typical post-fermented dark tea, which is increasingly popular for its unique flavour quality and excellent health benefits [1]. Its production technology is an intangible cultural heritage of China and has been inscribed on the UNESCO Representative List of the Intangible Cultural Heritage of Humanity. In 2023, dark tea products represented by Fu brick tea rode on the wave of growth, achieving an output value of CNY 31.04 billion (USD 4.286 billion), an increase of CNY 4.18 billion (USD 578 million) from the previous year, which represented the largest growth among the six major tea categories of dry and gross tea, with the second largest share of the output value after that of green tea and black tea (data from Tea China Marketing Association, https://www.ctma.com.cn/, accessed on 12 April 2024). However, the microbial fermentation process of Fu brick tea not only produces a pleasant “fungus flower” aroma but also “stale”, “sour”, and other undesirable odours [2]; simultaneously, as the Fu brick tea is stored for a longer time, it is more likely that the tea will be damaged [3]. At the same time, as the storage time of Fu brick tea goes on, especially the changes in storage environment condition factors, such as temperature, light, and humidity, will further affect the aroma characteristics of Fu brick tea, which will have a crucial impact on the production and consumption of Fu brick tea [4]. In this instance, co-fermenting Fu brick tea with mulberry leaves [5], moringa leaves [6], citrus peels [7], and other food and medicinal plants is not only an industrial invention but also a novel approach to successfully enhance the distinctive aroma of Fu brick tea.

Currently, there are relatively few studies on the characteristic attributes of the aroma of traditional tea with mulberry leaves, moringa leaves, and citrus peels. The studies that have been conducted have shown that the fung flower aroma of Fu brick tea is composed of 1-octanol and 1-heptadecanol with stale aroma attributes, methyl salicylate, acetophenone, and benzyl alcohol with green aroma attributes, 2-pentylfuran with roast aroma attributes, and geranyl acetone and β-violets with floral and fruity aroma attributes [8,9]. It is worth noting that the addition of Moringa leaves during the production of Fu brick tea can effectively contribute to the increase in the contents of aldehydes, ketones, and alcohols, as well as endow Fu brick tea with a rich floral and fruity aroma and the sweetness attributes of compounds such as 2,6,6-trimethyl-1-cyclohexene-1-carboxaldehyde and 3-buten-2-one, whereas traditional Fu brick tea only has the fatty aroma attribute of (E)-2-octenal [6,10]. For example, the addition of citrus peel to pu-erh tea improves the flavour quality of pu-erh tea with the odour-active components in citrus peel, giving citrus pu-erh tea a unique and harmonious flavour [7].

However, little research has been carried out on the impact of the addition of mulberry leaves to Fu brick tea on its aroma characterisation attributes. Importantly, China is not only the country with the largest mulberry planting area in the world but also the country with the largest number of mulberry varieties, and it is very rich in mulberry germplasm resources [11]. In particular, mulberry leaves are known as the king of plants, rich in unsaturated fatty acids, proteins, polyphenols, alkaloids, polysaccharides, γ-aminobutyric acid, and phytosterols and other nutrients and active ingredients, with hypoglycemic and hypolipidemic effects, lowering of serum cholesterol, scavenging of oxygen radicals, antibacterial and antiviral effects, and so on, which can prevent and control hypertension, diabetes mellitus, atherosclerosis, obesity, and other chronic diseases [12,13]. Therefore, processing mulberry leaves into Fu brick tea can not only make full use of mulberry leaf resources to meet the growing demand for functional foods but also explore its effect on key odour-active compounds of Fu brick tea and its characteristic aroma attributes.

In order to identify the aroma contributors in complex MTs and FTs, headspace solid–phase microextraction coupled with gas chromatography–mass spectrometry (HS-SPME-GC-MS) has been used in recent years to extract, isolate, and characterise the aroma from tea, which has become a widespread and effective method due to the advantages of its simple equipment, ease of operation, less sample consumption, shorter processing time, and the absence of the use of organic solvents [14,15,16]. Sensory evaluation is a set of techniques to accurately measure human perception of the whole flavour of a food product and is considered to be a very valuable method for analysing key odour-active compounds in relation to aroma character attributes [17,18,19]. However, aroma components exhibit different odour thresholds and concentrations, and only a few compounds are major contributors to the overall aroma, and these compounds are considered to be important key odourants [20]. The key odour compounds between them can be accurately screened and calculated using chemometrics and the odour activity value (OAV) [21,22,23]. This multifaceted approach helps to reveal the key odour components and assess their contribution in MTs versus FTs.

Therefore, the objectives of this study were to (a) analyse the aroma profile attributes of MTs and FTs based on sensory evaluation; (b) identify the key odour compounds that form the aroma profile attributes of MTs; and (c) elucidate the relationship between the aroma compounds of MTs and the sensory attributes. This study is of great significance for the in-depth understanding of the mechanism of aroma feature formation during the production of MTs, as well as a guide for the development of functional foods related to them.

## 2. Materials and Methods

### 2.1. Tea Sample

Nine tea samples with four MFs and five FTs were collected from Yun Tian Ge Tea Co. in Anhua County, Hunan Province, China. The samples were made in September 2022 according to the traditional Fu brick tea processing method. The samples were collected, transported back to be sealed, and stored in a 5 °C refrigerator for GC-MS analysis and aroma evaluation.

### 2.2. Chemicals

The analytical purity of the following reagents is >98%. The commercial sources of the standard compounds were as follows: geraniol, linalool, methyl salicylate, and nerolidol, purchased from TCI Reagent, Shanghai, China. Ethyl decanoate (J&K Scientific, Beijing, China) was used as an internal standard. The internal standard (ethyl decanoate) was added to a headspace vial for semiquantification of volatile compounds. Linear retention indices (LRIs) were determined using n-alkanes C_7_–C_40_ (Sigma-Aldrich Supelco, Shanghai, China).

### 2.3. Sensory Evaluation and Quantitative Descriptive Analysis (QDA) 

The samples from MTs and FTs were subjected to sensory evaluation, called quantitative descriptive analysis, by six professionals (three men and three women, with an average age of 35 years) from the Key Laboratory of the Ministry of Education in Tea Science, Hunan Agricultural University [24,25]. All panellists were previously trained in identifying different aroma attributes and were trained in identifying, describing, and quantifying the intensity of aroma characteristics of different Fu brick teas. The aroma profiles of MTs and FTs were finally identified and summarised through discussion.

Finally, we chose 6 aroma attributes to describe the overall aroma characteristics of MTs and FTs: “floral and fruity”, “green”, “fatty aroma”, “sweet”, “stale”, and “roasted” [2,26]. In accordance with the dark tea brewing standards described independently in GB/T 23776-2018 “Tea Sensory Review Methods”, 3 g of tea was brewed with 150 mL of boiling water for 5 min, and then, the tea broth was filtered [2]. The expert members rated the tea samples according to their aroma attributes using a scale of 0 to 5, with 0 meaning imperceptible, 1 perceptible, 2 weak, 3 moderate, 4 strong, and 5 very strong. Parallel tests were conducted three times.

### 2.4. HS-SPME Extraction of Tea Aroma

We weighed 0.5 g (accurate to 0.001 g) of the ground homogeneous sample in a 20 mL headspace extraction flask, added 10 mL of distilled water at 80 °C, and added 10 μL of ethyl decanoate (10 mg/L) as the internal standard. Subsequently, it was equilibrated in a water bath at 80 °C for 10 min to evaporate the volatile components fully. Then, the SPME needle was inserted into the extraction vial, and after fixing the extraction handle, the fibre tip was pushed out (extraction fibre tip type 50/30 μm DVB/CAR/PDMS, polydimethylsiloxane and divinylbenzene, Supelco, Bellefonte, PA, USA), the headspace adsorption was maintained at 80 °C for 30 min, followed by rapid insertion of the extraction head into the GC injection port and desorption at 250 °C for 5 min, and then the volatile compounds were used for GC-MS analysis.

### 2.5. Tea Aroma Determination by GC-MS

The prepared HS-SPME volatile compounds were analysed using an HP 7890B-5977A GC-MS coupled instrument purchased from Agilent Technologies (Santa Clara, CA, USA) with an HP-5MS column (30 × 0.25 mm, 0.25 um). Helium (He, purity > 99.999%) was used as the carrier gas, and the analysis of the HS-SPME was carried out without shunt injection, while the temperature of the injection port was 250 °C, and the heating procedure was as follows: the initial temperature was 40 °C, maintained for 3 min, and then warmed up to 210 °C with 3 °C/min, maintained for 1 min, and warmed up to 260 °C with 10 °C/min, and the sample volume was 1 μL. The flow rate was 1.0 mL/min, and the injection volume was 1 μL. The ionisation mode of mass spectrometry was EI mode, the ionisation energy was 70 eV, the temperature of the ion source was 220 °C, and the scanning range of the ion fragmentation was *m*/*z* 35~650.

### 2.6. Identification and Determination of Volatile Compounds

The National Institute of Standards and Technology (NIST) mass spectrometry database was used to search for compounds with >80% similarity, and the retention index (RI) of volatile compounds was calculated in combination with the n-alkane series C_7_–C_40_. Finally, the volatile compounds were identified by comparison with the RI in the database. Compound odour descriptions were determined using the Flavor odour library (https://www.nlm.nih.gov/, accessed on 22 March 2024). The relative content of volatiles was calculated from the internal standard (mg/L).

### 2.7. Calculation of Odour Activity Value (OAV)

The OAV is used to assess the contribution of volatile compounds to tea aroma. Volatile compounds with an OAV > 1 are considered aroma-active and play an important role in the aroma profile of tea [27,28].

### 2.8. Statistical Analysis

Data were analysed using Excel software (Excel 2016) (Addinsoft, New York, NY, USA) and Origin 2021 (Origin Lab, Northampton, MA, USA). TBtools software v2.096 (https://github.com/CJ-Chen/TBtools (accessed on 27 March 2024), China) was used for heatmap and cluster creation. OPLS-DA analyses were performed by SIMCA 14.1 (Umetrics, Umea, Sweden). ChemDraw 20.0 (CambridgeSoft, Cambridge, MA, USA) and Vision 2021 (Microsoft, Redmond, WA, USA) were used to draw the generation pathways and flowcharts of the compounds, respectively. The results of the experiments were repeated three times, and the data were expressed as mean ± standard deviation.

## 3. Results

### 3.1. Sensory Evaluation

**Figure 1:**
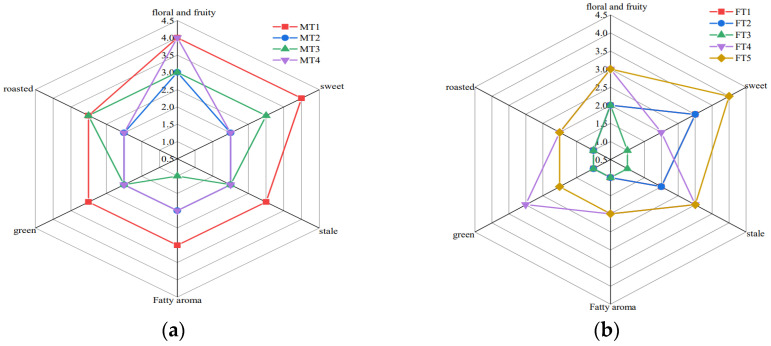
Radar charts of aroma profiles of MTs and FTs: (**a**) radar chart of aroma profiles of MTs; (**b**) radar chart of aroma profiles of FTs.

A traditional sensory quality evaluation was conducted to determine the difference in flavour quality between MTs and FTs. The results showed that MFs had a dominant floral and fruity aroma that fluctuated (Figure 1a), while FTs presented sweet aroma attributes (Figure 1b). Among the MTs, MT1 and MT4 had strong and long-lasting floral and fruity aroma attributes, with MT1 also carrying a strong sweet aroma attribute. MT2 and MT3 had mainly floral and fruity flavour attributes, with other aromatic attributes not standing out. 

In FTs, FT5 had a strong sweet aroma attribute, with floral and fruity and stale aroma attributes being stronger than the other aroma types. FT4 had mainly floral and fruity, green, and stale flavour attributes, while FT2 had mainly sweet flavour attributes. The aromas in FT1 and FT3 were not very obvious. These results indicate that the floral and fruity aroma in MTs is more prominent, while the aroma of FTs is dispersed and less intense.

### 3.2. Analysis of Volatile Components of MTs and FTs by HS-SPME-MS-GC

**Figure 2:**
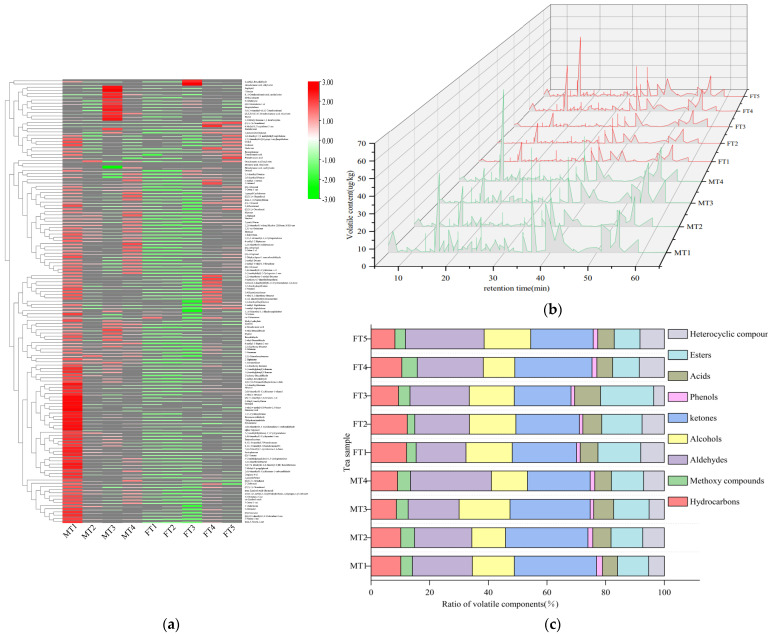
Clustering thermogram (**a**), aroma fingerprint (**b**), and stacking histogram (**c**) of volatile components in MTs and FTs.

We analysed the volatile components in MTs and FTs to compare the differences between them. We used HS-SPME-GC-MS for the analysis and identified a total of 139 volatile components. We created a clustering heat map to visualise and analyse the components (Figure 2a). Our findings showed that the volatile components in MTs were significantly different from FTs. MT1 had significantly more volatile components than the other MTs. On the other hand, FT1, FT2, and FT3 generally had low levels. Overall, MTs had more volatile components than FTs.

We also determined the 3D fingerprints of MTs and FTs based on the peak retention time, concentration, and other relevant parameters of the aroma components (Figure 2b). The types of aroma components were similar in both, but the contents of each type of substance were different. We identified 22 hydrocarbons, 6 methoxy compounds, 28 aldehydes, 19 alcohols, 32 ketones, 4 phenols, 4 acids, 12 esters, and 12 heterocycles. The main volatile components in both were aldehydes, ketones, and alcohols. In MTs, the relative contents of these components were 21.25%, 26.21%, and 13.86%, respectively. In FTs, these components were 26.30%, 27.62%, and 18.40%, respectively. We found that hydrocarbons and esters had higher proportions in FTs than in MTs, which were 9.49%, 11.11%, 13.11%, and 16.09% in MTs and FTs, respectively. Acids and heterocyclic substances were also higher in FTs than in MTs, 8.08% and 9.01% in FTs and 5.95% and 6.25% in MTs, respectively (Figure 2c).

**Figure 3:**
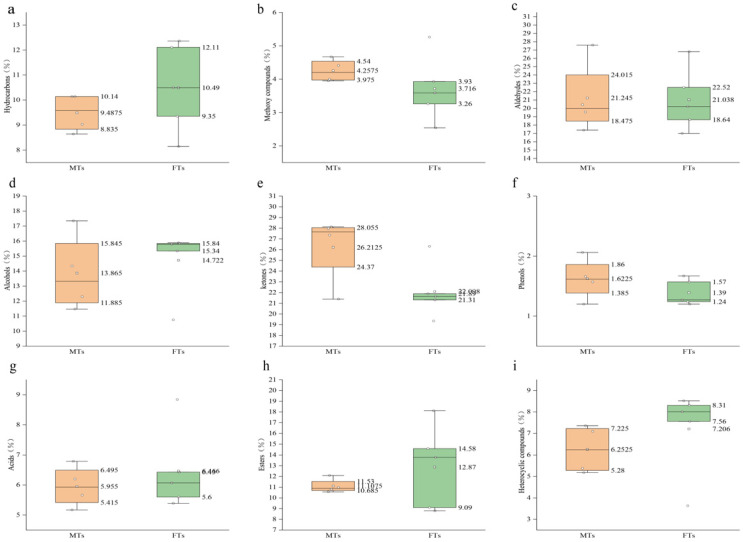
Analysis of different volatile compositional attributes of MTs and FTs. Note: (**a**) hydrocarbons; (**b**) methoxy compounds; (**c**) aldehydes; (**d**) alcohols; (**e**) ketones; (**f**) phenols; (**g**) acids; (**h**) esters; (**i**) heterocyclic compounds.

In Figure 3, it is shown that Fu brick tea contains more hydrocarbons, esters, and heterocyclic compounds in FTs than in MTs. Hydrocarbons make up a relatively small proportion of the overall volatile composition of Fu brick tea and make a limited contribution to its aroma. Esters, on the other hand, have pleasant aroma attributes such as sweet, green, and musky flavours. Methyl salicylate and dihydroactinidiolide are two examples of esters that contribute less to the overall aroma of Fu brick tea due to their higher thresholds and lower values of odour activity. Heterocyclic compounds are formed by the Meladic reaction of the tea processing to form aroma compounds, and 2-pentyl-Furan carries a floral, fruity, and fatty aroma which has a role in the formation of the flavour qualities of MTs and FTs. 

Methoxy compounds and phenolic and acid compounds are consistent in both MTs and FTs. They have a synergistic effect on the formation of stale and fatty flavour qualities of Fu brick tea. Aldehydes, ketones, and alcohols also play an important role in the aroma attributes of Fu brick tea. They are more abundant in MTs than in FTs. For example, Benzaldehyde, 2-ethyl-1-Hexanol, 6-methylhept-5-en-2-one, Cedrol, (E)-3,7,11,15-tetramethylhexadec-2-en-1-ol, and other compounds are important contributors to the formation of floral and fruity, sweet, and green aroma attributes in MTs and FTs.

### 3.3. Screening for Characteristic Volatile Components

**Figure 4:**
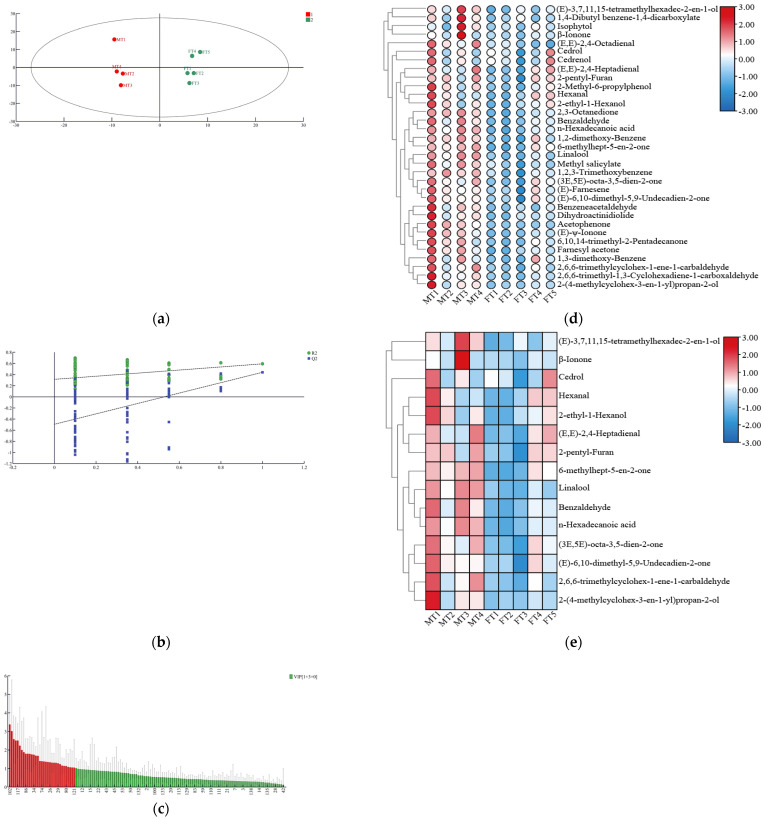
Validation of characteristic volatile compounds of MTs and FTs. (**a**) OPLS-DA scatter plots of MTs and FTs; (**b**) OPLS-DA model validation; (**c**) significance of (VIP) variables among 139 volatile fractions in MTs and FTs (red columns highlighting volatile fractions with VIP > 1); (**d**) thermograms of 33 differentially accumulated volatile compounds in MTs and FTs (VIP > 1); (**e**) thermograms of 15 differentially accumulated volatile compounds in MTs and FTs (OVA > 1 and VIP > 1).

The correlation between the levels of volatile components and their odours in MTs and FTs is more difficult to screen than the characteristic components affecting both. A new multivariate statistical analysis method, OPLS-DA, was used, which can screen the key components affecting the aroma characteristics of MTs and FTs [29,30]. The model was used to differentiate the aroma characteristics of MTs and FTs and performed well in terms of model fit (R^2^Y = 0.97) and predictive power (Q^2^ = 0.72) without overfitting, and MTs and FTs were divided into two groups (Figure 4a,b). Typically, when the VIP (variable importance factor) is greater than 1, aroma substances are considered key components for distinguishing between the two.

The OPLS-DA model identified 33 volatile components with VIP > 1 and *p* < 0.05, including (E)-Farnesene, 1,2-dimethoxy-Benzene, 1,3-dimethoxy-Benzene, 1,2,3-Trimethoxybenzene, Benzaldehyde, Hexanal, 2,6,6-trimethyl-1,3-Cyclohexadiene-1-carboxaldehyde, Linalool, Cedrenol, 2,3-Octanedione, Isophytol, (E,E)-2,4-Heptadienal, Benzeneacetaldehyde, (E)-3,7,11,15-tetramethylhexadec-2-en-1-ol, β-Ionone, (E,E)-2,4-Octadienal, 2-(4-methylcyclohex-3-en-1-yl) propan-2-ol, 2,6,6-trimethylcyclohex-1-ene-1-carbaldehyde, Cedrol, 2-ethyl-1-Hexanol, (E)-ψ-Ionone, 6-methylhept-5-en-2-one, 6,10,14-trimethyl-2-Pentadecanone, 2-pentyl-Furan, (3E,5E)-octa-3,5-dien-2-one, 2-Methyl-6-propylphenol, n-Hexadecanoic acid, (E)-6,10-dimethyl-5,9-Undecadien-2-one, Acetophenone, Farnesyl acetone, Methyl salicylate, Dihydroactinidiolide, and 1,4-Dibutyl benzene-1,4-dicarboxylate (Figure 4c). The volatile components were found to be significantly different in MTs and FTs based on a clustered heat map visualisation (Figure 4d). The content of the 33 volatile components was somewhat higher in MTs than in FTs. These findings suggest that these volatile components play a crucial role in determining the differences in aroma characterisation attributes between MTs and FTs.

The aroma components of MTs and FTs cannot be determined based on their content. It is the higher OAV value that gives the characteristic aroma of the tea leaves. The contribution of individual aromas to the overall aroma of tea can be evaluated by calculating the OAV. The OAV is considered to have a certain influence on the aroma of tea when the OAV is >1, and it is considered to make a great contribution to the overall aroma of tea when the OAV is >10 [2]. To determine the OAVs of the characteristic aroma components of MTs and FTs, researchers calculated the threshold values and attribute descriptions of aroma components reported in the literature. The results showed that a total of 15 volatiles with OAV > 1 were identified, which significantly contributed to the characteristic attributes of the aroma of MTs and FTs (Table 1). These key aroma compounds include Hexanal, Benzaldehyde, β-Ionone, Cedrol, 2,6,6-trimethylcyclohex-1-ene-1-carbaldehyde, 2-pentyl-Furan, 2-(4-methylcyclohex-3-en-1-yl) propan-2-ol, n-Hexadecanoic acid, (3E,5E)-octa-3,5-dien-2-one, (E)-3,7,11,15-tetramethylhexadec-2-en-1-ol, Linalool, 6-methylhept-5-en-2-one, (E,E)-2,4-Heptadienal, (E)-6,10-dimethyl-5,9-Undecadien-2-one, and 2-ethyl-1-Hexanol.

### 3.4. Differences in Aroma Attribute Characteristics between MTs and FTs

To visually represent the differences in aroma components between MTs and FTs, a heat map visualisation was used to analyse 15 aroma components with OAV > 1 (Figure 4e). The results indicated that the aroma components in MTs and FTs were significantly different. MTs contained 13 aroma components with OAV > 1, including Hexanal, 6-methylhept-5-en-2-one, and (E)-6,10-dimethyl-5,9-Undecadien-2-one with green attributes, Benzaldehyde with roasted aroma attributes, 2-ethyl-1-Hexanol and 2-pentyl-Furan with a floral and fruity aroma, Linalool with a floral and fruity aroma, 2-(4-methylcyclohex-3-en-1-yl) propan-2-ol and (E)-3,7,11,15-tetramethylhexadecadien-2-en-l-ol, and β-Ionone with a floral aroma, Cedrol with sweet aroma attributes, (3E,5E)-octa-3,5-dien-2-one with floral and green attributes, and n-Hexadecanoic acid with fatty aroma attributes. On the other hand, FTs contained 10 aroma constituents with an OAV > 1, including Benzaldehyde, 2-ethyl-1-Hexanol, 6-methylhept-5-en-2-one, n-Hexadecanoic acid, 2-(4-methylcyclohex-3-en-1-yl) propan-2-ol, (3E,5E)-octa-3,5-dien-2-one, Cedrol, 2-pentyl-Furan, (E)-3,7,11,15-tetramethylhexadec-2-en-1-ol, and (E)-6,10-dimethyl-5,9-Undecadien-2-one.

Seven aroma components in MTs are considered important, as they have OAVs greater than 10. These aroma components include Benzaldehyde, 2-(4-methylcyclohex-3-en-1-yl) propan-2-ol, (E)-3,7,11,15-tetramethylhexadec-2-en-1-ol, (E)-6,10-dimethyl-5,9-undecadien-2-one, Cedrol, (3E,5E)-octa-3,5-dien-2-one, and 6-methylhept-5-en-2-one. On the other hand, only one aroma component in FTs (Cedrol with a sweet aroma attribute) is considered important, as it has an OAV greater than 10. Compounds such as MTs with a floral and fruity aroma and the green properties of Benzaldehyde, (E)-6,10-dimethyl-5,9-Undecadien-2-one, 2-(4-methylcyclohex-3-en-1-yl) propan-2-ol, 6-methylhept-5-en-2-one, (3E,5E)-octa-3,5-dien-2-one, and (E)-3,7,11,15-tetramethylhexadec-2-en-1-ol have higher OAVs than their OAVs in FTs. These compounds are important for the formation of the significant floral and fruity aroma, as well as the green aroma attribute characteristics of MTs.

## 4. Discussion

Volatile Aroma is an important criterion for evaluating the tea quality of tea leaves [33]. The selection of key active aroma compounds affecting MTs and FTs plays a crucial role in the overall aroma evaluation of both. The difference in flavour quality between MTs and FTs was determined by traditional sensory quality evaluation [17,18,19]. Under the same processing technique, MTs had strong floral and fruity and green aroma attributes, while FTs showed sweet aroma attributes.

**Figure 5:**
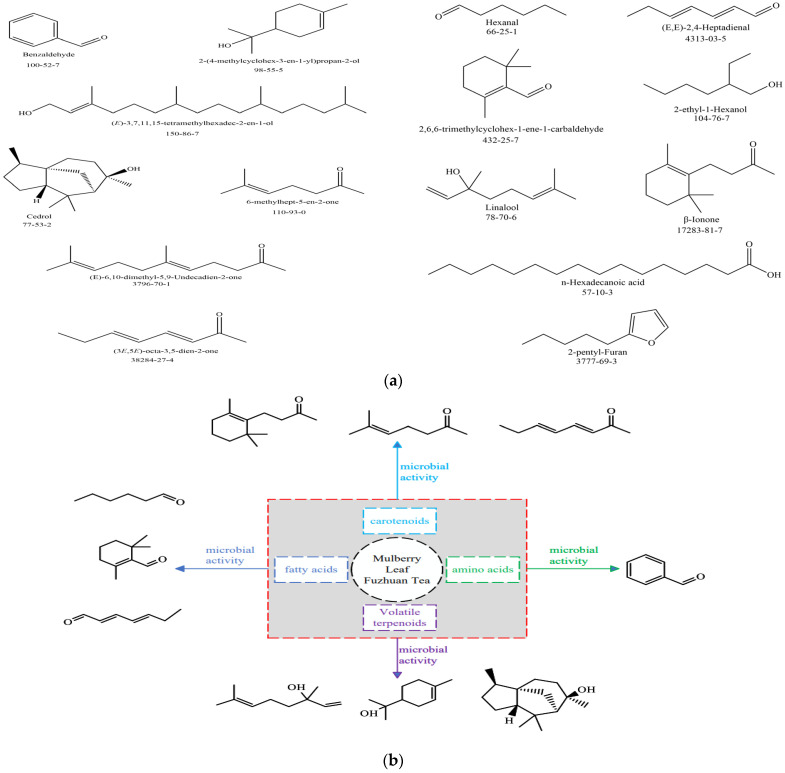
Key odour substances for high aroma intensity identified in MTs. (**a**) Chemical structure of key odour substances; (**b**) possible pathways for the formation of key odour substances.

In order to identify the key volatile constituent differences between MTs and FTs, a profiling of the characteristic constituents of the two Fu brick teas was carried out based on the HS-SPME-MS-GC technique [34]. A total of 139 volatile components were identified. Thirty-three volatile components had a VIP > 1. Among them, seven volatile components had OAVs > 10, namely (E)-3,7,11,15-tetramethylhexadec-2-en-1-ol, Cedrol, Benzaldehyde, 2-(4-methylcyclohex-3-en-1-yl) propan-2-ol, (3E,5E)-octa-3,5-dien-2-one, 6-methylhept-5-en-2-one, and (E)-6,10-dimethyl-5,9-Undecadien-2-one, most of which have floral and fruity, sweet, green, and other aroma attributes [27] and are found in MTs, which were all higher in OAVs than those in FTs. Thus, it is believed that the above substances are the main active substances that make MTs exhibit stronger floral and fruity aromas than FTs (Figure 5). The differences may be attributed to the fact that a certain bioactive component in mulberry leaves has a promotional effect on the nutrient content as well as microbial growth during the fermentation process of Fu brick tea, which in turn can effectively improve the aroma quality of Fu brick tea [35].

Cedrol, 2-(4-methylcyclohex-3-en-1-yl) propan-2-ol, and (E)-3,7,11,15-tetramethylhexadec-2-en-1-ol are produced by cleavage of the glycosylation bonds of β-D-glucoside and β-sakuranoside [36]. Relevant studies have shown that the contents of Cedrol, 2-(4-methylcyclohex-3-en-1-yl) propan-2-ol, and (E)-3,7,11,15-tetramethylhexadec-2-en-1-ol were increased by the microbial dominant strain of Aspergillus during the fermentation of Pu-erh tea. The key enzymes for the formation of volatile terpene alcohol aroma components were sakuranosidase and glucosidase [37]. Carotenoid derivatives (CDVs) derived from various carotenoids, including β-carotene, α-carotene, Phytoene, lutein, and lycopene [38], can be produced by microbial enzymatic oxidative degradation under the action of carotenoid cleavage dioxygenases to give rise to (E)-6,10-dimethyl-5,9-Undecadien-2-one, 6-methylhept-5-en-2-one, and (3E,5E)-octa-3,5-dien-2-one. Experimentally, we demonstrated that (E)-6,10-dimethyl-5,9-Undecadien-2-one originates from the degradation of carotenoids, and M Ibdah proved that (E)-6,10-dimethyl-5,9-Undecadien-2-one is a product of expressed CmCCD1, which induces oxidative degradation at C9-C10 of the carbon chain of Phytoene [39,40]. Fatty acid derivatives (FADVs) can be produced by the action of lipoxygenase (LOX) and hydroperoxide lyase (HPL) produced by microbial activity to produce C6–C9 aldehydes, such as 2,6,6-trimethylcyclohexane-1-ene-1-carbaldehyde with fruity flavouring attributes, Hexanal with green attributes, (E,E)-2,4-Heptadienal, and others [41]. During the fermentation of Fu brick tea, amino acid derivatives (AADVs) significantly increase the content of phenylalanine-derived benzaldehyde and methionine-derived methionine through microbial enzymatic oxidative degradation [42]. In addition to enzymatic oxidative degradation, Strecker degradation of amino acids is considered to be the main reason for the significant production of roasted aroma benzaldehyde during fermentation [43].

Studies have shown that mulberry leaves are rich in active nutrient components such as unsaturated fatty acids, proteins, polyphenols, alkaloids, polysaccharides, γ-aminobutyric acid, and phytosterols [13]. Under the action of microbial enzymes, they will replenish the compounds degraded during the fermentation process, which provides sufficient substrates for Fu brick tea fermentation [38]. Relevant studies have shown that during tea fermentation, free amino acids are metabolised into corresponding volatiles by enzymatic and Strecker degradation, which theoretically reduces the level of free amino acids [44]. On the other hand, free amino acids are replenished through protein degradation [45]. Thus, free amino acid levels are in a dynamic mode of change during fermentation. In conclusion, the addition of mulberry leaves to Fu brick tea has a very important role in improving the flavour quality of tea. However, how the bioactive components in mulberry leaves affect the enzyme activities of microorganisms during the fermentation of Fu brick tea needs to be further investigated.

## 5. Conclusions

In summary, this study identified the main volatile compounds in MTs and FTs. MTs have very obvious “flowery and fruity” and “green” flavour attributes, while FTs have “sweet” flavour attributes. At the same time, seven important aroma markers with OAVs > 10 were screened out, which play a very important role in the aroma of MTs and FTs. In addition, this is the first time that mulberry leaves were added to Fu brick tea for co-production. The research results are of great significance in understanding the formation mechanism of key aroma characterisation compounds in MTs and FTs, which will guide the development of functional foods related to them.

## Figures and Tables

**Table 1:** Odour activity values (OAVs) of key compounds in MTs and FTs (OAV > 1).

No.	Volatile Compound	OT (ug/L) ^a^	OAV									
			MT1	MT2	MT3	MT4	FT1	FT2	FT3	FT4	FT5	Odour Description ^b^
1	Hexanal	4.5	2.77	1.12	0.57	0.73	0.35	0.32	0.57	1.36	1.37	green
2	Benzaldehyde	0.75	15.20	6.74	13.51	8.64	3.78	3.23	4.24	7.26	6.70	roasted
3	(E,E)-2,4-Heptadienal	56	0.83	0.44	0.40	1.10	0.24	0.27	0.19	0.64	0.86	green, fatty aroma
4	2,6,6-trimethylcyclohex-1-ene-1-carbaldehyde	5	1.06	0.39	0.52	0.82	0.30	0.27	0.19	0.51	0.32	floral and fruity
5	2-ethyl-1-Hexanol	0.4	17.05	8.94	4.16	8.16	2.73	2.61	5.26	6.69	8.48	floral and fruity, fatty aroma
6	Linalool	6	1.29	0.81	1.37	1.26	0.47	0.35	0.33	0.68	0.47	floral and fruity
7	2-(4-methylcyclohex-3-en-1-yl) propan-2-ol	0.3	29.43	6.38	10.66	10.70	4.38	5.40	4.77	7.28	6.15	floral
8	Cedrol	0.5	34.67	9.52	17.70	8.91	15.47	12.43	3.82	9.57	29.04	sweet
9	(E)-3,7,11,15-tetramethylhexadec-2-en-1-ol	0.46	16.81	12.07	32.32	17.93	6.41	7.39	13.56	8.34	12.96	floral
10	6-methylhept-5-en-2-one	0.3	12.10	9.00	12.27	13.52	2.22	1.85	2.57	9.83	8.36	green
11	(3E,5E)-octa-3,5-dien-2-one	0.5	24.73	11.73	9.37	17.70	5.17	4.48	2.62	14.21	10.42	floral, green
12	β-Ionone	1	1.69	0.88	11.52	0.79	0.75	0.64	0.25	1.24	0.97	floral
13	(E)-6,10-dimethyl-5,9-Undecadien-2-one	3	20.39	10.22	9.86	10.03	5.92	6.26	2.63	11.66	7.96	green
14	n-Hexadecanoic acid	10	2.50	1.85	2.58	2.30	1.18	1.08	1.26	1.71	1.69	fatty aroma
15	2-pentyl-Furan	4.8	3.09	2.98	1.32	3.76	1.05	0.90	0.32	2.82	2.73	floral and fruity, fatty aroma

^a^: OT, odour thresholds in water. Odor thresholds in water were obtained from [31,32]; ^b^: odour description found in the literature using database (Flavornet; https://pubchem.ncbi.nlm.nih.gov/).

## Data Availability

The original contributions presented in the study are included in the article, further inquiries can be directed to the corresponding author.
